# Personalized Federated Learning Scheme for Autonomous Driving Based on Correlated Differential Privacy

**DOI:** 10.3390/s25010178

**Published:** 2024-12-31

**Authors:** Yuan Tian, Yanfeng Shi, Yue Zhang, Qikun Tian

**Affiliations:** 1 School of Computer Engineering, Nanjing Institute of Technology, Nanjing 211167, China; ytian@njit.edu.cn; 2 Electrical Engineering and Computer Science (EECS), KTH Royal Institute of Technology, 10044 Stockholm, Sweden; qikunt@kth.se

**Keywords:** federated learning, correlated differential privacy, autonomous driving

## Abstract

In the era of big data, advanced data processing devices and smart sensors greatly benefit us in many areas. As for each individual user, data sharing can be an essential part of the process of data collection and transmission. However, the issue of constant attacks on data privacy arouses huge concerns among the public. This work proposes a personalized federated learning method associated with correlated differential privacy for autonomous driving. First, instead of transmitting raw data to the server following collection, a device that employs federated learning can perform calculations to obtain the training model at each node. Second, we specifically perform a correlated classification analysis to encrypt data that share high relevance, which can minimize the system cost. Then, correlated differential privacy is utilized to achieve the preservation of data privacy before sharing. In contrast to the traditional differential privacy, the proposed solution guarantees enhanced privacy to meet the demands of customization. The experimental results show that our scheme is more refined in terms of user heterogeneity and the utility of data than others without violating privacy.

## 1. Introduction

Over the past few decades, with the rapid development of information technology and artificial intelligence, the frequency of data use has increased dramatically. A large amount of data are constantly circulated between users and devices as they interact with the world on a daily basis to obtain personalized services, which indeed brings great convenience. However, these personal data are highly likely to be exposed to hidden hackers who aim to violate users’ privacy, thereby raising public awareness of data protection, sharing, and utility to a higher level. General Data Protection Regulation (GDPR) emphasizes the strict protection of personal data, requiring appropriate technical and organizational measures to be taken when processing personal data to prevent data breaches or unauthorized access.

In the automotive industry, since the introduction of autonomous vehicles (AVs), built-in cameras, radar, and other sensors have been applied to collect a large amount of data during the driving process for the continuous analysis and improvement of autonomous driving, thus creating a better driving experience. Additionally, it can also assist in epidemic tracking. Nevertheless, sensitive data related to users’ privacy may pose a certain threat. For example, when self-driving cars transmit road information data during driving, their sensors inevitably capture the images of the surroundings on the street. The collected information may be hacked by unscrupulous individuals for illegal purposes before or during transmission to the server. Worse still, inappropriate data storage and extraction methods used by car manufacturers may lead to the leakage of individual sensitive information.

**Figure 1:**
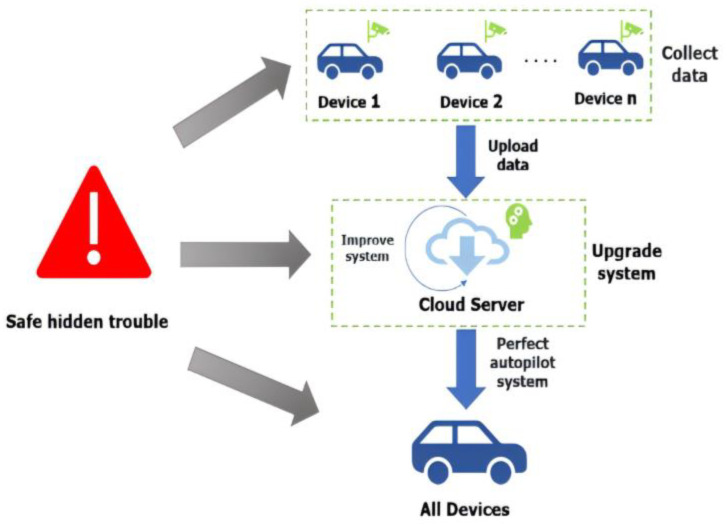
Potential hidden troubles.

For devices such as autonomous vehicles, after the collection of raw data, these data are usually transmitted to the server for aggregation. The data transmission process is also known as data sharing. Direct data sharing may be attacked during transmission, resulting in privacy leakage. During data aggregation, the central server aggregates the model parameters received from various devices to generate a global model. Since data aggregation occurs in distributed systems, data volatility is an issue that cannot be ignored in distributed systems. For example, when the network signal is poor or the battery is low, some devices may not be able to upload updated model parameters in a timely manner, which can affect the training results of the global model. Data sharing, which is beneficial to device development, may instead be difficult to complete or even fail due to data volatility if misdetected. Federated learning (FL) [1] is urgently needed to solve this problem. However, several issues need to be considered before this technology can be successfully applied. Merely sharing the learned and updated data in a single global model through training and analysis seems to be unable to capture the habits of individual users. In addition, devices such as autonomous vehicles vary in performance, which may affect the learning efficiency, resulting in delays or omissions in updates. Moreover, security cannot be ignored when the server receives trained data. Since differences between users, even those with similar connections, need to be considered, multitask learning [2] generates customized models. At this moment, even if the targeted data are encrypted, the information associated with that data could still probably be obtained by the attacker, who can consequently infer the targeted data. Thus, it seems that striking a trade-off between the degree of data privacy protection and data accuracy is a major challenge in the field of data protection. Hence, the question of how to prevent data from being stolen without violating the rules of data collection is extremely crucial nowadays. Employing differential privacy (DP) to add noise to query results through Gaussian, Laplacian, or exponential mechanisms is an approach that can be used to safeguard data collection. Differential privacy protects data privacy by adding random noise, which theoretically provides strong protection; however, the effectiveness of this method largely depends on the level of noise introduced. Although promising, the existing research focusing on single sets cannot meet the data requirements in a correlated environment [3]. This methodology may present potential challenges, as shown in Figure 1.

- The performance of devices varies from one to another, making the current privacy scheme unsuitable for application in this field. Further research should be carried out to develop a more sophisticated scheme to address the differences between devices.
- The amount of correlated sensitivity will increase as the number of correlated datasets grows, which requires the addition of a large amount of noise. This will reduce the protection effect of related datasets.
- A vast amount of information implies that the number of individual-purpose queries doubles or even triples. Meanwhile, the privacy budget must be divided into smaller parts, which may be the reason for the increased noise in each query.

Through hiding information in a non-IID [4] dataset, the privacy of data exhibiting strong coupling relations can be preserved: when encountering a large number of queries, as each user has a different privacy budget and demands a different level of privacy protection, the use of correlated sensitivity produces less noise, thus mitigating the loss of data utility and also saving on privacy costs [5]. Correlated differential privacy [6] is therefore required in our article, which allows us to reduce the addition of redundant noise by analyzing data attributes when processing relevant data. However, the setting of the noise level directly affects the balance between privacy protection and data availability. For highly correlated data, too little noise may increase the risk of privacy leakage, while too much noise can seriously affect the practicality of the data. Therefore, we must carefully set the noise level to ensure that the data remain useful while providing sufficient privacy protection. As the raw data accumulate in each device, according to the performance of devices, the correlated differential privacy will generate a correlated analysis [7] to ensure the diversity and applicability of data transmission, which enhances the credibility of these data with lessened interference from the outside world.

To address the above issues, we propose an enhanced user privacy preservation scheme which offers a viable customized training model and takes data heterogeneity into account. This is a scheme that considers device conditions while guaranteeing data privacy at the time when data are transmitted that have already been learned. First, due to the fear of insufficient data preservation during the sharing period, we assume that each device has embedded computing power and is able to compute the raw data once data acquisition is complete. Following a series of self-learning and optimization processes, different individual training models are formed and then these models are uploaded to the server, where a collaboration is coordinated between devices so that these models can be improved based on data from others. Second, the correlated analysis of data based on correlated differential privacy promises to provide data of different types, along with security detection, before delivery to the server with the help of a mixed dataset. The aim of our design is to make sure all the training models cannot be decrypted to obtain more details under any attack from potential malicious users. In conclusion, the main contributions of this article are as follows:

- Personalized federated learning is employed in autonomous vehicles based on a correlated differential privacy.
- Correlated differential privacy is used to ensure that data with strong relevance are encrypted in view of privacy interventions.
- A correlated classification algorithm is provided to achieve more cost-efficient privacy protection.
- We conduct simulations on privacy preservation and the relationship between the number of noises needed and attribute-correlated analysis. The performance evaluation indicates that the data with similar attributes after encryption exhibit the efficiency and cost–benefit attributes of the whole system.

## 2. Related Work

Over the past years, as the growing concern about the protection of data privacy and security spread among the public, the concept of federated learning was further strengthened. This enables data fusion and modeling without compromising internal data. Federated learning is a kind of distributed machine learning [7,8]. Compared to existing distributed machine learning methods, worker nodes are not stable. Since data stored on each worker node are not independent and are identically distributed, the balance of raw data may be affected. Despite this, users will have more control over their device and data than before, and this method is still widely used in machine learning to deal with the issue of privacy.

For the moment, federated learning constitutes a trinity that combines machine learning, distributed frameworks, and privacy-preserving technologies. Modern distributed devices such as autonomous vehicles, wearables, smartphones, and IoT sensors often generate a large amount of data every second and minute. A diverse range of tasks can be solved through different learning models which are developed using the abundant data gathered. There are several interesting applications, including transportation networks [9], personalized language models [10], and situational awareness for driving autonomously [11]. Edge devices with enhanced computational power are used to decentralize the data and propagate the network computation to the client. From the perspective of privacy preservation, it is also quite effective.

A significant amount of research has been conducted on FL recently [12]. Xiang et al. [13] rely on FL to protect mobile edge computing, and Xiao et al. [14] select vehicles while keeping the system’s overall cost to a minimum. FL and differential privacy are widely discussed in papers [15,16]. Likewise, Truex et al. [17] employ secure multiparty computation and centralized differential privacy to prevent inference from both messages exchanged in the model training process. Sadly, the privacy budget affecting the performance of FL is not taken into consideration when managing analysis. A privacy-preserving FL approach is presented by Hu et al. [18] to enable customized models to learn more effectively. Being associated with a Gaussian mechanism and a centralized DP mechanism, such a mechanism can better protect the privacy of the model. In our article, we expect to train multiple personalized models of disparate autonomous vehicles. Here, multitask learning mixes two categories together to fit the model relationship between tasks either randomly unclarified or as a priori.

For FL, Yang et al. proposed a model poisoning attack method, which was a unique way to shuffle and adjust model parameters. Since the local data and training models of clients are invisible to the server, malicious clients can submit gradients that are deliberately designed to disrupt the accuracy of the model [16]. To mitigate malicious behavior from clients, it can use encryption or blockchain technologies to resist poisoning attack [19]. In this work, we use encryption processing.

With the advent of the era of big data, people have become increasingly aware of the need to protect their privacy. The privacy issue arises from the inability of a server or device to maintain the confidentiality of the data or models in a distributed system coordinated by a central server. The presence of certain security risks during the use of data can bring about the leakage of user privacy.

Several privacy preservation techniques, such as data masking [20], anonymization [21], differential privacy [22], and homomorphic encryption [23], have been frequently used in the past to secure privacy. In the last few years, most of the literature has only investigated differential privacy. Most existing work on differential privacy assumes that there is no relationship among data. There is, however, no guarantee that data privacy can be protected well when traditional differential privacy is used to protect data because most of the data delivered are correlated. However, correlated differential privacy and correlated analysis can address this concern.

Data in the dataset are usually correlated to some degree and each piece of data is related to the others. Kifer and Machanavajjhala [24] were the first authors to argue that the correlation between data will lead to the privacy leakage of individuals. In [25], they proposed a new privacy framework called “Pufferfish”, which can be used to interpret correlations between data. Although Chen et al. [26] considered the correlation between the data and the way they are presented by multiplying the number of records of relevant data by the global sensitivity. This is an approach that introduces too much noise into the query, which can make the results less useful. As for [3], Zhu et al. named a relevant data release mechanism by defining the correlated sensitivity. Such a mechanism is more suitable for the data release of correlated datasets on an iterative basis.

In the correlation analysis, Zhang et al. [27] proposed a method of associating data by using IP addresses in network traffic. This method is mainly used for correlation analysis in network data. Zhu et al. [3] calculated the gathered data by employing attribute analysis, time interval analysis, and Pearson correlation analysis, but this way of analyzing the correlation between data in correlation analysis is not comprehensive enough. However, in the context of our research, the differences in attributes and features of the dataset make these traditional methods not fully applicable. Therefore, we propose a new correlation analysis method—correlation classification analysis. In our research, data do not exist in isolation, but rather as a collection of data integrated through multiple attributes and features. When conducting data correlation analysis, some datasets have specific attributes that only exist in that dataset and do not appear in other datasets. Therefore, the correlation analysis of these data should take into account this specific attribute and only encrypt and protect attributes with high levels of correlation. For example, when analyzing collected car data, certain attributes (such as the average speed of vehicles) may only exist in specific datasets. At this point, data with specific attributes can be selectively encrypted according to privacy requirements, while ignoring other data. In this way, privacy protection can be applied in a more targeted way while avoiding unnecessary computational overhead. In this article, the correlation of data is represented by a correlation matrix, which distinguishes different correlation sizes by defining a threshold. Compared to global sensitivity methods, the use of correlated sensitivity can effectively reduce redundant noise and more accurately protect data privacy. In this way, we avoid the problem of the increased noise caused by traditional global sensitivity methods and make the privacy protection of the system more precise and efficient.

Compared with recent related existing schemes, our scheme supports three properties, as shown in Table 1, simultaneously: data relevance, personalization, and privacy preservation.

## 3. Preliminaries

### 3.1. Multitask Learning

**Definition 1.**  *(multitask learning): for m-related learning tasks ${\left\{T_{t}\right\}}_{t=1}^{m}$ , the goal of multitask learning is to improve the learning performance of the model for one task of them.*

### 3.2. Correlation

To comprehensively measure data correlation, we define the correlation as follows.

**Definition 2.**  *(correlation): If two records are correlated with each other, these two records are $b_{i}$ *and* $b_{j},$ respectively. The correlation between them can be represented by $\gamma_{ij}$* *, where $\gamma_{ij}\in \left[-1,1\right]$* *and $\left|\gamma_{ij}\right|\geq \gamma_{0}$* *, with $\gamma_{0}$* *being the threshold value of the correlation.*

**Corollary 1.** 
$When\;\left|\gamma_{ij}\right|=1$ *, we assume that records $b_{1}$* *and $b_{2}$* *are completely correlated. When $\left|\gamma_{ij}\right|=0$* *, we assume that records $b_{i}$* *and $b_{j}$* *are completely uncorrelated. When $\gamma_{ij}<0$* *, we assume that records $b_{i}$* *and $b_{j}$* *are negatively uncorrelated. When $\gamma_{ij}>0$* *, we assume that records $b_{i}$* *and $b_{j}$* *are positively uncorrelated.*

When $\left|\gamma_{ij}\right|$ tends to 1, it means that $b_{i}$ and $b_{j}$ are strongly correlated. If we delete or encrypt one of them, it will have great impact on the other. But when $\left|\gamma_{ij}\right|$ tends to 0, it means that $b_{i}$ and $b_{j}$ are weakly correlated. If we try to delete or encrypt, the chances are low that it will influence the other.

Based on the correlation γ between the data, we can generate a correlation matrix ω:

(1) $$\omega =\left(\begin{array}{cccc} \gamma_{11} & \gamma_{12} & \ldots & \gamma_{1n}\\ \gamma_{21} & \gamma_{22} & \ldots & \gamma_{2n}\\ \ldots & \ldots & \ldots & \ldots \\ \gamma_{n1} & \gamma_{n2} & \ldots & \gamma_{nn} \end{array}\right)$$

In the matrix ω, the elements on the diagonal represent the correlation between its own parts, and they are perfectly correlated, i.e., $\gamma_{11}$, $\gamma_{22}$, ⋯ $\gamma_{nn}$ are all 1. As the matrix ω is symmetrical, that is $\gamma_{ij}=\gamma_{ji}$, we only need half a matrix to complete what we are looking for:

(2) $$\omega =\left(\begin{array}{cccc} \gamma_{11} & \gamma_{12} & \ldots & \gamma_{1n}\\ \ldots & \gamma_{22} & \ldots & \gamma_{2n}\\ \ldots & \ldots & \ldots & \ldots \\ \ldots & \ldots & \ldots & \gamma_{nn} \end{array}\right)$$

### 3.3. Federated Learning

In this section, we first illustrate how personalized federated learning is achieved; then, correlated differential privacy is used to provide a privacy guarantee.

Personalized federated learning can be described as the problem of multitask learning

(3) $$\begin{array}{c} \underset{W,\Omega }{\min}G\left(W,\Omega \right):\left\{\overset{m}{\underset{t=1}{\sum }}\overset{n_{t}}{\underset{i=1}{\sum }}l_{t}\left(w_{t}^{T}x_{t}^{i},y_{t}^{i}\right)+ℜ\left(W,\Omega \right)\right\}\\ s.t.\Omega ≻=0,tr\left(\Omega \right)=1 \end{array}$$

which contains two components.

1. Empirical loss function: In the system, all the data are presented as a feature matrix $X:=diag\left(X_{1},...,X_{m}\right)\in {\mathbb{R}}^{md\times n}$ and m is the total number of local devices which are distributed remotely. Here, we take the activities of autonomous vehicles as an example. Each single node (vehicle) $t\in \left[m\right]$ is usually distinguished from the others. Denote by $W:=diag\left(W_{1},...,W_{m}\right)\in {\mathbb{R}}^{d\times n}$. The matrix for collective model parameters, in which $w_{t}\in {\mathbb{R}}^{d}$, belongs to separate parameters for device t. The i-th column of data $X_{t}\in {\mathbb{R}}^{d\times n},$ where $n_{t}$ stands for the sum of the training sample in device t’s database, refers to a feature vector $x_{t}^{i}\in {\mathbb{R}}^{d}$. Moreover, label $y_{t}^{i}$ is the t-th element of vector $y_{t}$ in a set. Joining these three variables through convex loss functions, $l_{t}$ is the first term of measurement of the empirical loss of all training samples.

2. Learning task relationship function: the matrix $\Omega \in {\mathbb{R}}^{m\times m}$ constructs the relationship model among different tasks, which can either be processed in order of priority or estimated simultaneously. The formulation $ℜ\left(W,\Omega \right)$ in Formula (3) is defined as follows:

(4) $$ℜ\left(W,\Omega \right)=\lambda tr\left(W\Omega^{-1}W^{T}\right)$$

With the constant λ>0, which is a regularization parameter, a second term of measurement of learning task relationship is used.

### 3.4. Differential Privacy

Differential privacy provides privacy protection for two adjacent datasets with a very rigorous theoretical basis [30]. Suppose that there are two datasets known as x and x’, respectively. Provided that the two datasets have the same cardinality between them and only one sample is different between them, then x and x’ are said to be adjacent datasets. The query function can be expressed as $f\left(x\right):x\to R$. The randomization mechanism M accompanied by differential privacy represents the final result obtained by adding a deterministic query result $f\left(x\right)$ to a random noise [31].

**Definition 3.**  *(ε-differential privacy) [31]: ε-Differential privacy is brought forward for a mechanism M in terms of a pair of adjacent datasets x and x’ for any output set S, it satisfies the following:*

(5) $$Pr[M\left(x\right)\in S]\leq e^{\varepsilon }Pr[M\left(x’\right)\in S’]$$

*where ε is privacy budget. As ε is becoming smaller, the level of privacy protection is higher. When ε=0, the probability of output between x and x’ is the same for M*.

Global sensitivity is the measurement of the maximum change in view of the query result $f\left(x\right)$ when randomly deleting a dataset x from the sample. Global sensitivity is related to mechanism M.

**Definition 4.**  (ε*-global sensitivity).* *For $f\left(x\right):x\to R$, the global sensitivity of $f\left(x\right)$* *is defined in* [22] *as follows:*

(6) $$GS=\underset{x,\;\;x^{\prime }}{max}\|f\left(x\right)-f\left(x^{\prime }\right){\|}_{1}$$

*where* || *is Manhattan distance, denoted by*

(7) $$\|a\|=\left|a_{1}\right|+\left|a_{2}\right|+\ldots +\left|a_{n}\right|$$

The function max works for any adjacent dataset x and x’; the global sensitivity here is the largest Manhattan distance. Since you can enter as many as you like, max is needed to obtain the maximized number.

When applying differential privacy to privacy protection, the data to be processed generally fall into two broad categories, one numerical and one non-numerical. Different mechanisms are to be used for these two types of data. For numerical data, the Laplace or Gaussian mechanism is generally used, and differential privacy can be achieved by adding random noise to the result obtained. For non-numerical data, the exponential mechanism is generally used.

The Laplace mechanism provides ε-differential privacy, and for function $f\left(x\right):x\to R$, the Laplace mechanism is defined below [32].

**Definition 5.**  *(Laplace mechanism)*.

(8) $$M\left(x\right)=f\left(x\right)+Laplace\left(\frac{GS_{f}}{\varepsilon }\right)$$

*The exponential mechanism pairs with score function* $f\left(x,\psi \right)$*. This score function indicates how good an output scheme* ψ* is for the query *f [22]*. The exponential mechanism is defined as* [33].

**Definition 6.**  *(exponential mechanism)*.

(9) $$M\left(x\right)=\{return\;\psi \;with\;the\;probability\;\propto exp(\frac{\varepsilon f\left(x,\psi \right)}{2GS_{f}})\}$$

**Definition 7.**  *(correlated sensitivity)*.

(10) $$CS_{i}=\overset{n}{\underset{j=0}{\sum }}\left|\gamma_{ij}\right|\left(\|f\left(x^{j}\right)-f\left(x^{-j}\right){\|}_{1}\right)$$

(11) $$CS_{q}=\underset{i\in q}{max}=\left(CS_{i}\right)$$

*where *$CS_{i}$* is record sensitivity. This denotes the size of the impact on all records in a dataset x when deleting record* $b_{i}$. $CS_{q}\;$*is the correlated sensitivity and is the maximum value of the record sensitivity; q is the set of records for all records of query* f  [22]*. After obtaining the correlated sensitivity, the final result of the noise added by correlated differential privacy is expressed as follows:*

(12) $$f^{\prime }\left(x\right)=f\left(x\right)+Laplace\left(\frac{CS_{q}}{\varepsilon }\right)$$

*in which* $f^{\prime }\left(x\right)$ *indicates the result after adding noise.*

## 4. The Proposed System

**Figure 2:**
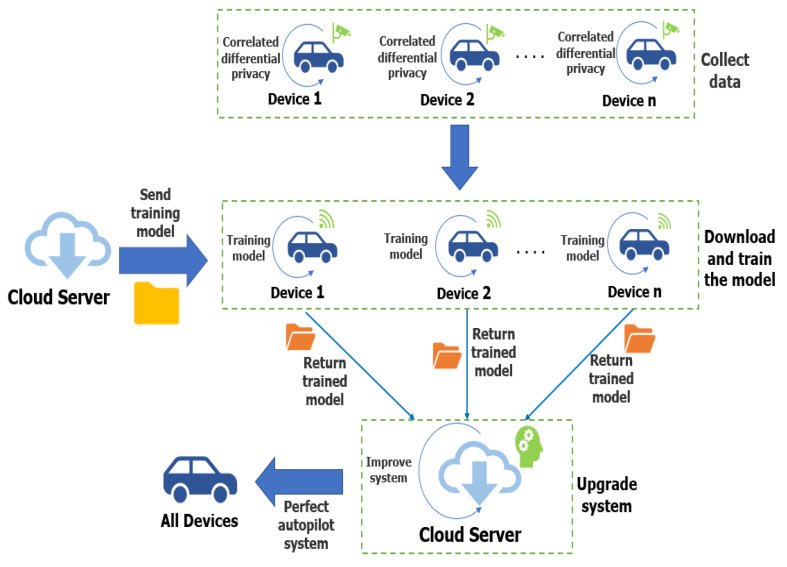
Overall architecture.

In this section, we propose a model in which a personalized federated learning system associated with correlated differential privacy is applied in autonomous driving, which is shown in Figure 2.

### 4.1. System Mode

(1) Federated learning is a distributed machine learning method that aims to train models locally through multiple devices (such as smart phones, autonomous vehicles, etc.) without transmitting the original data to the central server. This method ensures the privacy of user data, as each device only needs to transmit updated model parameters without involving actual data. Although traditional federated learning methods can protect user data privacy, in heterogeneous device environments, training effectiveness and model performance may be affected due to differences in device performance and data distribution. As each varies in performance, computation capability, and battery capacity under different network and road conditions, autonomous vehicles in our setting are heterogeneous. Though equipped with federated learning system to share trained models, these vehicles may lack the capability to tell the differences in habits between users. What is worse, under most circumstances, this method seems to be unable to detect minor differences among users who are close, minimizing the acquired data utility. Multitask learning [2] can be a good method to learn personalized models to address this problem.

(2) Although federated learning protects data privacy through local model training, the transmission of model parameters may still face potential privacy leakage risks. In particular, malicious attackers may infer the user’s raw data by analyzing model updates. To address this issue, we introduced correlated differential privacy techniques to further enhance the privacy protection capability of federated learning systems. Normally, differential privacy is achieved by adding noise through Gaussian, Laplacian, or exponential mechanisms. However, some improvements are made in our work. The correlation between similar data is assessed so that the possibility of data being leaked can be greatly reduced. As a result, correlated differential privacy with correlated data analysis can help to ensure privacy before releasing data models. To sum up, in our proposed personalized federated learning system, the role of correlated differential privacy is reflected in the following ways:

- Data encryption processing: before the local model update transmission, correlated differential privacy is used to add noise to highly correlated data, ensuring that sensitive information contained in the model parameters is not easily inferred by attackers.
- Dynamic noise adjustment: Correlated differential privacy dynamically adjusts the degree of noise addition through the analysis of data correlation in order to achieve a balance between privacy protection and data practicality. This method not only enhances privacy protection, but also reduces unnecessary noise introduction and maintains model performance.

### 4.2. Privacy Model

(1) Central servers sometimes are not completely trustworthy. They could violate users’ privacy by observing updated training models. For example, a server or a malicious user can recover the trained data using a model inversion attack [34], or a membership inference attack [31] detecting associated data can also keep the user’s personal privacy in danger.

(2) An updated model, which is trained through federal learning, will be further processed by classifying the correlated data in the model and then using correlated differential privacy to add noise to the data that show high degrees of correlation. Due to this, the chance of user’s personal data being hacked or leaked is relatively low.

(3) As different users have different demands for the level of privacy protection of their personal information, users can choose different levels of privacy protection according to their privacy protection needs, achieving personalized privacy protection.

## 5. The Proposed Algorithm

### 5.1. Personalized Federated Learning

Though we find it hard to optimize all the variables in Formula (3), an alternating optimization procedure [35] can be used to tackle the previous problem regarding the changes between W and Ω. Specifically, we first optimize Ω in objective function with fixed W and then optimize W when Ω is fixed.

1. Optimize Ω with fixed W:

When W is fixed, the function needed to solve the problem here can be simplified as follows:

(13) $$\begin{array}{c} \underset{\Omega }{\min}G\left(\Omega \right):\left\{\lambda tr\left(\Omega^{-1}WW^{T}\right)\right\}\\ s.t.\Omega ≻=0,tr\left(\Omega \right)=1 \end{array}$$

Analyzing the equation

(14) $$\begin{array}{l} tr\left(\Omega^{-1}B\right)=tr\left(\Omega^{-1}B\right)tr\left(\Omega \right)\\ \;\;\;\;=tr\left(\left(\Omega^{-\frac{1}{2}}B^{\frac{1}{2}}\right)\left(B^{\frac{1}{2}}\Omega^{-\frac{1}{2}}\right)\right)tr\left(\Omega^{\frac{1}{2}}\Omega^{\frac{1}{2}}\right)\\ \;\;\;\;\geq {\left(tr\left(\Omega^{-\frac{1}{2}}B^{\frac{1}{2}}\Omega^{\frac{1}{2}}\right)\right)}^{2}={\left(tr\left(B^{\frac{1}{2}}\right)\right)}^{2} \end{array}$$

$B=WW^{T}$. We can infer that the first equation is equal due to the last constraint in formula (13) and the last inequality always holds due to the Cauchy–Schwarz inequality for the Frobenius norm. Additionally, if and only if $\Omega^{-\frac{1}{2}}B^{\frac{1}{2}}=a\Omega^{\frac{1}{2}}$ for constant a and $tr\left(\Omega \right)=1$, the left side of the Equation (14) reaches its minimum value ${\left(tr(B^{\frac{1}{2}})\right)}^{2}$. 

Obviously, the solution for Ω is acquired:

(15) $$\Omega =\frac{{\left(W^{T}W\right)}^{\frac{1}{2}}}{tr(\left(W^{T}W{)}^{\frac{1}{2}}\right)}$$

2. Optimize W with fixed Ω:

Under this condition, we start by extending works on distributed devices to realize primal dual optimization, the subproblem becomes

(16) $$\underset{\Omega }{min}G\left(W\right):\left\{\overset{m}{\underset{t=1}{\sum }}\overset{n_{t}}{\underset{i=1}{\sum }}l_{t}\left(w_{t}^{T}x_{t}^{i},y_{t}^{i}\right)+\lambda tr\left(W\Omega^{-1}W^{T}\right)\right\}$$

Considering the fact that the dual formulation of (3) has the property of separability, allowing it to divide the global problem into distributed subproblems, we consequently conclude the following:

(17) $$\underset{\Omega }{min}D\left(\alpha \right):\left\{\overset{m}{\underset{t=1}{\sum }}\overset{n_{t}}{\underset{i=1}{\sum }}l_{t}^{*}\left(-\alpha_{t}^{i}\right)+{ℜ}^{*}\left(X\alpha \right)\right\}$$

where $l_{t}^{*}$ and ${ℜ}^{*}$ are the conjugate dual functions of $l_{t}$ and ℜ, respectively, i.e., $l_{t}^{*}\left(-\alpha \right)=\max_{v}\{-\alpha v-l\left(v\right)\}$ and $\hat{\Omega }:=\Omega ⊗I_{d\times d}\in {\mathbb{R}}^{md\times md}$. The element $\alpha_{t}^{i}$ derived from column vector $\alpha \in {\mathbb{R}}^{n}$ is the dual variable for the sample point $\left(x_{t}^{i},y_{t}^{i}\right)$.

To solve the Formula (3), we develop an iterative search algorithm based on the approximation of local data quadratic subproblems of the dual Formula (17).

We define the i-th subproblem:

(18) $$\underset{△\alpha_{t}}{min}{ϒ}_{t}^{\beta^{\prime }}\left(\Delta \alpha_{t};v_{t},\alpha_{t}\right):\left\{\overset{n_{t}}{\underset{i=1}{\sum }}l_{t}^{i}\left(-\alpha_{t}^{i}-\Delta \alpha_{t}^{i}\right)\right\}\;+w_{t}\left(\alpha \right)X_{t}\Delta \alpha_{t}+\frac{\beta^{\prime }}{2}\|X_{t}\Delta \alpha_{t}{\|}_{\hat{\Omega }}^{2}+c\left(\alpha \right)$$

where $c\left(\alpha \right):=\frac{1}{m}{ℜ}^{*}\left(X\alpha \right)$. Among these subproblems, $\Delta \alpha_{t}\in {\mathbb{R}}^{n_{t}}$ is the t-th block vector to dual variables in α of each single device t. The dual variables α can be obtained in this optimization via $w\left(\alpha \right):=\nabla {ℜ}^{*}\left(X\alpha \right)$, a column vector concatenated from m blocks of primal variables, with $w_{t}\left(\alpha \right)$ being the t-th block. What is more, $\hat{\Omega }\in {\mathbb{R}}^{d\times d}$ is the t-th diagonal block of the symmetric positive definite matrix $M\in {\mathbb{R}}^{md\times md}$ when Ω is fixed. It is also important to know that when vector ν=Xα, $v_{t}\in {\mathbb{R}}^{d}$ is the t-th block and only needs to be returned after each iteration. Finally, β’ is the correction parameter in the subproblems. The detailed steps are shown in Algorithm 1.
**Algorithm 1:** Personalized Federated Learning**Input:** Datasets $X_{t}$, m devices, inner global iteration number *J*, outer global iteration number *L* and correction parameter *β’*.
1: **Initialization: **column vector α←0, v←0;
2: **for**
l=1→L **do**
3: **for**
j=1→J **do**
4:  **for** all *m* devices in parallel **do**
5:   update the variable $\alpha_{t}\leftarrow \alpha_{t}+\Delta \alpha_{t}$
6:  return $\Delta v_{t}=X_{t}\Delta \alpha_{t}$
7:  update the vector $v_{t}\leftarrow v_{t}+\Delta v_{t}$
8:  **end**
9:  Update *W* to the server
10: **end**
11: Update Ω with α from the $w\left(\alpha \right)$
12: **end**

The proposed personalized federated learning algorithm can balance privacy protection and model performance. Personalized federated learning disperses the model training process across different devices, avoiding the direct transmission of raw data and reducing the risk of data leakage. At the same time, personalized federated learning allows each device to update the model locally and then aggregate it to the server. This method ensures privacy protection while effectively improving model performance. Furthermore, it has high flexibility. This algorithm supports the adjustment of the training process based on the computing power of different devices and user needs, and adapts to various distributed environments, such as heterogeneous devices and unstable network conditions, by optimizing training parameters. Moreover, it can reduce the burden of data transmission. Compared to traditional centralized learning methods, personalized federated learning reduces the amount of data that need to be transmitted to a central server, only transmitting model update parameters. This has significant advantages in situations with large data volumes or poor network conditions. Although the algorithm has high flexibility, its performance may decrease when dealing with devices with high heterogeneity.

### 5.2. Correlated Analysis

By reviewing the relevant literature, we learned about several commonly used correlated analysis methods, such as attribute analysis [36], time interval analysis [37], and Pearson analysis [36]. But when these correlated analysis methods are applied to a dataset, the correlated analysis of the dataset and the division of attributes are not suitable enough, and so we propose a new correlated analysis method, which is called correlated classification analysis. When conducting correlation analysis, we not only use traditional attribute analysis methods, but also combine various data attributes and features to improve the accuracy of correlation analysis. For example, in the processing of autonomous driving data, we divide data into different groups based on correlation, and only encrypt when there is strong correlation between the data. In this way, the system can ensure that only highly correlated data are encrypted, while avoiding the unnecessary encryption of low-correlation data, reducing computational and storage overhead. For each attribute in the dataset, we set different thresholds based on the correlation of the data. When setting these thresholds, we use feature-based correlation metrics and multi-party correlation metrics. The selection of these thresholds is not only based on the nature of the data itself, but also takes into account the different privacy protection needs. In this way, we can dynamically adjust the encryption level of data to meet different privacy protection needs and reduce the burden on the system when processing data.

The data collected do not exist as points, but constitute a collection of data that integrates many kinds of data, associated with many attributes. When analyzing the correlation between data, some datasets have attributes that only exist within the dataset and do not exist in other datasets, so the data in this dataset with this attribute have no correlation with other datasets and can therefore be excluded from data correlation analysis. When encrypting data, these data can be selectively encrypted according to the emphasis that individual attaches to on privacy. For example, in Table 2, in the correlated analysis of the collected data, only C has the average speed while driving. As regards such attributes, these data can therefore be selectively ignored when performing correlated analysis and encrypting data of high relevance.

There are various approaches to correlated analysis, and even though we have proposed a new analysis method, the aim is to generate a correlated matrix ω by defining the threshold $\gamma_{0}$. If global sensitivity was used, this would lead to redundant noise in the results; thus, traditional global sensitivity cannot be adopted, and instead we use correlated sensitivity to solve this problem [36].

### 5.3. Correlated Classification Analysis (CCA)

In order to protect the privacy of the user’s data, a large amount of noise is often inevitably added to the raw data to ensure that they cannot be accessed by unscrupulous people. The disadvantage of doing this is that the introduction of large amounts of noise could lead to a significant reduction in the usefulness of the data. The ideal data privacy protection is one that ensures the usefulness of the data while also ensuring that the data are not accessible to unscrupulous persons.

Therefore, on the basis of correlated differential privacy, we conduct a deeper study on aspects such as correlation. A set of attribute-based analysis methods that can improve the utility of the data effectively by reducing the inclusion of redundant noise while maximizing the protection of data privacy is proposed at the same time. We store data by defining multiple arrays. The arrays are stored in different combinations depending on the size of the correlation. Users can personalize the level of protection according to their privacy budget. Users with high privacy requirements can choose to encrypt data in both high- and low-relevance arrays at the cost of reduced privacy utility. Users with low privacy requirements can choose to only encrypt a few high relevance arrays.

In Algorithm 2, k is the array used to store the data. The size of k can be assigned by how carefully the data are divided based on correlation size, and the more carefully the correlation size interval is divided, the larger the value of k will be.
**Algorithm 2:** Correlated Classification Analysis**Input:** Array for storing data k, he correlation size of the data ω, the thresholds that divide the correlation size intervals *c*, *d*, *e*
**Output: **$a\left[k\right]$
1: **for** int *n* = 1; *n* > *k*; *n*++ **do** 2:  String $a=‘a\left[’+n+‘\right]’$;
3:  String α[] = new String[n];
4:   **if**
 ω≥c **then**
5:     return $a\left[1\right]$
6:   **else if**
c>ω≥d **then**
7:     return $a\left[2\right]$
8:   **else if**
d>ω≥e **then**
9:     return $a\left[3\right]$
10:   **else if**
d>ω **then**
11:    return $a\left[4\right]$
12: **end**

ω is the correlation size of the data, while c,d,and e are the thresholds that divide the correlation size intervals. The selection of threshold values is based on subjective experience, but can refer to the feature-oriented correlation degree method or multiparty correlation degree method in reference [38]. The k and loop structure allows the creation of a corresponding number of arrays, after which the corresponding data are divided into the corresponding arrays according to the judgement of the size of ω, and finally the array with the loaded data is the output.

Here, we define a value p, which is the degree to which the client needs to encrypt data if the data are divided into 4 arrays of relevant sizes. When p=2, the user chooses to encrypt the data within the two arrays representing the greater relevance, which leaves the remaining two arrays representing the less relevant data unencrypted. Thus, we achieve a personalized encryption process that can greatly meet the privacy protection needs of each user.

**Figure 3:**
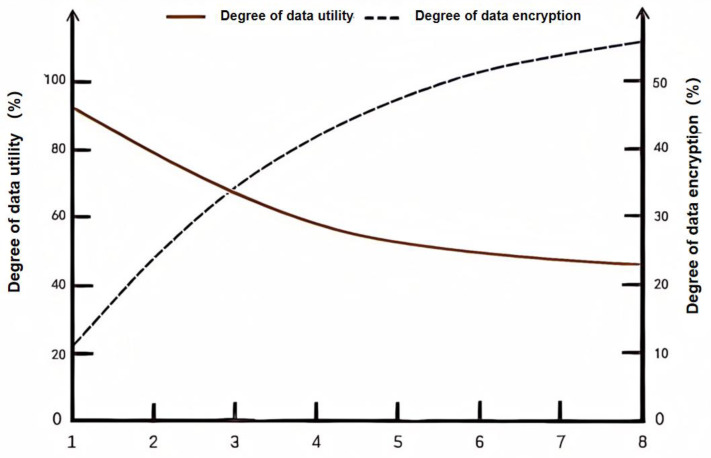
Degree of data utility vs. degree of data encryption.

Figure 3 illustrates the relationship between the degree of data utility and the degree of data encryption, varying from value p=1 to value p=8. These two lines show an apparent inverse relationship. We can conclude that the higher the level of data encryption, the lower the availability of data. When p=2, it means that the user chooses to encrypt the data within the two arrays representing the greater relevance. The data start to be encrypted with only 20% of total data. In the meantime, the data utility begins to fall, declining from 90% to nearly 70% of the total. When p=4, the degree of data encryption (above 40%) rises over the degree of data utility (nearly 25%), meaning that the greater the percentage the data encrypted, the lower the utility of data, which can decrease the chance of raw data being acquired by unscrupulous people. When p=6, the level of data encryption reaches the highest point—almost 50%—and the degree of data utility drops to the lowest level—23% in total—resulting in an enhanced level of data protection with reduced risk of being hacked by others.

The proposed CCA algorithm has stronger privacy protection. CCA encrypts highly correlated data by analyzing their correlation, reducing the possibility of potential attackers inferring the data. Compared to conventional differential privacy methods, it can more effectively protect privacy for relevant datasets, especially when dealing with highly correlated sensitive data. Additionally, it can reduce system overheads. By layering data with different correlations, this algorithm can reduce unnecessary noise and thus lower the computational and storage overheads of the system. This allows the algorithm to maintain high degrees of privacy protection without significantly reducing the practicality of the data. However, in large-scale datasets or multi-attribute data environments, the processing time may be extended. 

## 6. Performance Results and Analysis

In this section, we evaluate the performance of the proposed system in terms of privacy preservation and the relationship between the number of noises needed and whether to employ attribute-correlated differential privacy.

Experimental environment. We conducted experiments on a server equipped with an Intel Core (TM) i7-9750H processor and 128 GB of memory while using multiple edge devices that simulate autonomous vehicles. The device configuration includes a high-performance group (8-core CPU (central processing init), 16 GB of memory) and a low-performance group (4-core CPU, 8 GB of memory). The network environment is simulated under unstable conditions (latency of 50–200 ms, bandwidth of 10–50 Mbps). The experimental dataset contains real driving data collected from autonomous vehicles, covering attributes such as vehicle speed, position, and time.

Evaluation method. 1. To evaluate the effectiveness of privacy protection, we compared the data leakage risks before and after encryption. Without encryption protection, raw data are vulnerable to attacks from malicious users, leading to data leakage. To verify the effectiveness of our proposed differential privacy methods, we compared the data before and after encryption, and evaluated the risk of data leakage during the process of malicious attackers inferring the original data through model inference and other methods. By comparing the security of unencrypted data and encrypted data in experiments, we conclude that applying correlated differential privacy can effectively reduce the risk of data being obtained by criminals. 2. We evaluated the relationship between the utility of data and noise level. In the encryption process, the introduction of noise is inevitable, but excessive noise can affect the availability of data. Therefore, we analyzed the practicality of data under different levels of noise intensity. Specifically, we evaluated the relationship between the accuracy of encrypted data and the intensity of noise. We measured the validity of the data at different levels of noise and analyzed the extent to which the practicality of the data decreased with increasing noise. Through our experimental results, we demonstrated that a balance can be achieved between privacy protection and data availability. 3. We evaluated the effectiveness of data correlation analysis. We use relevant classification analysis algorithms to evaluate its effectiveness in reducing noise levels and system overhead. By comparing the data encryption situation when the relevant classification analysis is not applied and when the algorithm is applied, we found that centralized encryption of data with the same attributes could reduce the amount of noise required by the system, thereby reducing the privacy protection cost of the system. The experimental results show that the relevant classification analysis method effectively reduces redundant noise, improves system efficiency, and does not significantly reduce the practicality of data while maintaining a high level of privacy protection.

### 6.1. Performance Results

When delivering the data of car speed to the terminal server, instead of directly transmitting the raw statistics which are susceptibility to risks of intentional attack from anonymous users, we can apply the correlated differential privacy to encrypt these data to achieve a safer uploading process.

**Figure 4:**
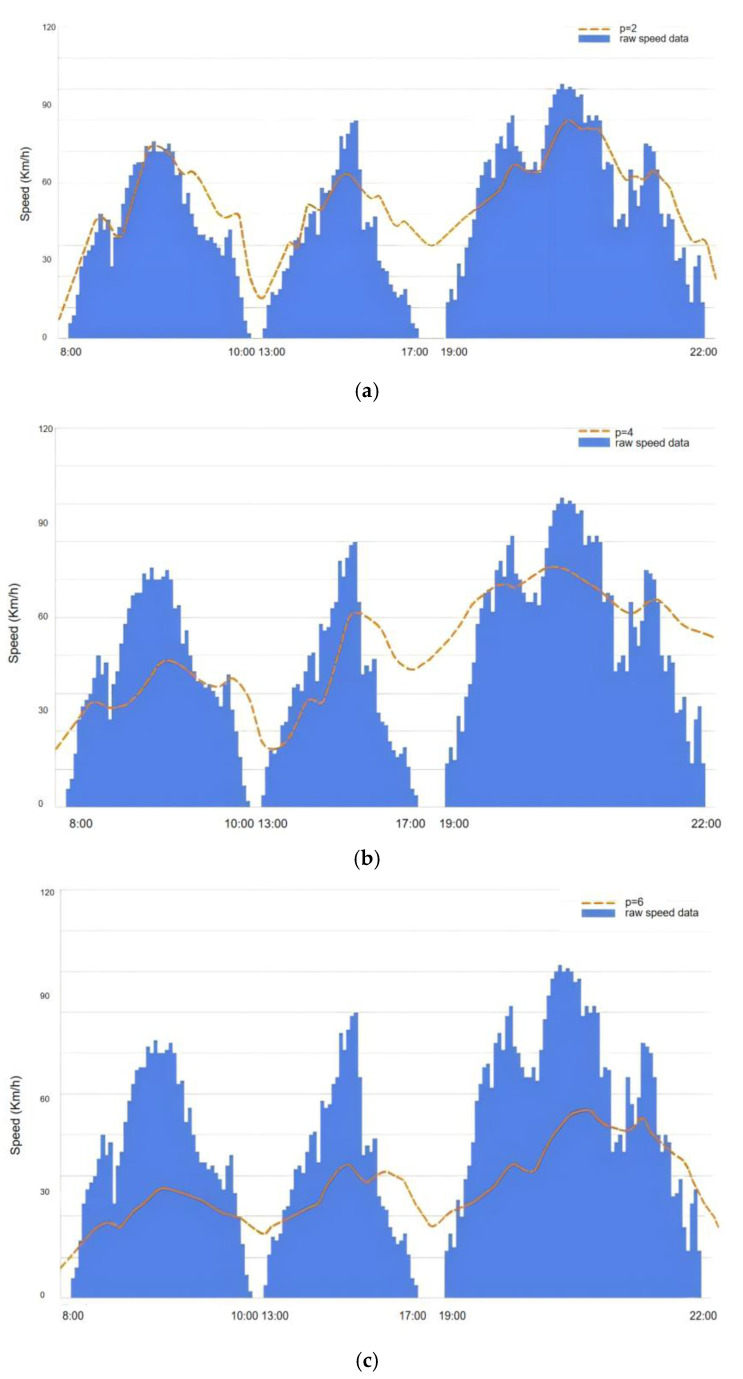
Performance of CCA when (**a**) p=2; (**b**) *p* = 4; and (**c**) *p* = 6.

Here, we take the data from a car during employment as an example. Figure 4 illustrates the data before the encryption and after the encryption. We can see clearly see that data on the fold line are much smaller than the original data. Normal data delivery without any protection procedures in terms of privacy can result in the data being stored by malicious servers in some way. Unlike common differential privacy techniques that help to preserve privacy, we use correlated differential privacy by analyzing the shared attributes between these data gathered. After the data are encrypted, chances are low that hackers could decipher the relevant data, enhancing the credibility of raw data. The result of the experiment of the proposed algorithm derives from the changing encryption value p, which varies from 2 to 6.

When p=2, the difference between these two means that data are collected by adding noises, leading to less data being violated by unscrupulous users. However, as these data encryption level varies with the car speed of a potential user, there is a danger that some data could still be acquired at some time. At two time intervals—8 a.m. to 10 a.m. and 1 p.m. to 5 p.m.—during a day, we can find that the car is being used and encrypted in order to transmit data to the server. Nevertheless, the interval between 10 a.m. and 1 p.m. matters a lot for the whole process. During such hours, data can be easily accessed without some protection as the car is not being used. Such is the case for the period between 7 p.m. and 10 p.m., when correlated differential privacy is not available.

When p comes to 4, the position of the fold line drops. The gap between encrypted data and raw data is reduced, meaning that the data are further encrypted on top of the original one. Data generated while the vehicle is in motion during the period of 8 a.m.–10 a.m., 1 p.m.–5 p.m., and 7 p.m.–10 p.m. can be adequately protected. And finally, the value p equals to 6 and the line drops even lower. Likewise, data from the three intervals depicted receive better protection in cases of leakage. It should be flexibly adjusted according to specific application scenarios to comply with GDPR’s requirements for privacy and security.

### 6.2. System Performance

**Figure 5:**
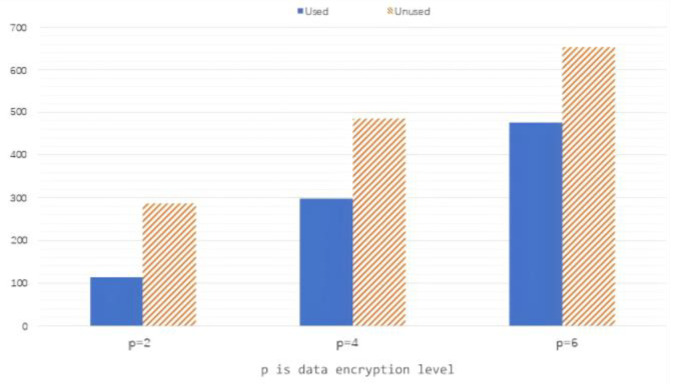
Number of noises needed.

When conducting the correlated analysis of data, some datasets from this car have attributes that only exist in a certain dataset, and other datasets do not have those attributes, so the data in this dataset with those attributes have no correlation with other datasets, meaning there is no need to encrypt these data. As we have mentioned above, correlated differential privacy based on certain attributes needs data to be added to query noises. Figure 5 depicts the relationship between the number of noises required and whether the correlated classification analysis has been applied or not for three conditions: p=2, p=4, and p=6.

The columns in the three comparison graphs show that a bunch of query noises are added to the system; however, we do not apply the correlated classification analysis of differential privacy that we suggested previously for each column on the right. Per the definition, under these circumstances, which normally involve privacy protection, there is no doubt that the number of noises increase dramatically. Consequently, referring to Figure 3, we can easily conclude that the applicability of data weakened further.

In contrast, the number of noises used when under the correlated classification analysis within differential privacy scheme is much lower than that without the scheme, as is shown on the left one of the comparison graphs. Data with the same attribute can be encrypted together, saving a great deal of system privacy cost. In this way, the number of noises needed is fewer than in the other one.

## 7. Conclusions

This paper proposes a solution with a privacy-preserving scheme using correlated differential privacy and personalized federated learning and applies it to the upgrading process of autonomous driving systems. An efficient and accurate privacy-preserving mechanism is obtained by encrypting highly correlated data before releasing the training model to the server. Furthermore, we also evaluated our solution based on a large amount of data and proved the feasibility of the scheme.

In future work, we will design an algorithm that achieves a more balanced combination of correlated differential privacy and personalized federated learning. We will also attempt to reduce the system cost and use minimal noise to achieve a better level of data privacy preservation. On the other hand, although we adopted a multi-level noise mechanism to some extent to resist attacks from malicious attackers, there is still room for further improvement in addressing robustness issues caused by device failures and other factors.

## Figures and Tables

**Table 1:** Property summary for recent related works.

Methods	Data Relevance	Personalization	Privacy Preservation
[12,18,19,20,21,26,28]	×	×	√
[15]	×	√	√
[3,22,23,24,25,29]	×	√	×
Our scheme	√	√	√

**Table 2:** Destination information.

Destination Information			
Destination	Location	Time of Arrival	Average Speed When Driving
A	X	9:00	
B	Y	12:00	
C	7	16:00	50 km/h
D	M	20:00	

## Data Availability

The data presented in this study are available on request from the corresponding author. The data are not publicly available due to privacy.

## Abbreviations

The following abbreviations are used in this manuscript:

GDPR	General Data Protection Regulation
CCA	correlated classification analysis
AVs	autonomous vehicles
FL	federated learning
DP	differential privacy
CPU	central processing unit
